# Brain Potentials Highlight Stronger Implicit Food Memory for Taste than Health and Context Associations

**DOI:** 10.1371/journal.pone.0154128

**Published:** 2016-05-23

**Authors:** Heleen R. Hoogeveen, Jacob Jolij, Gert J. ter Horst, Monicque M. Lorist

**Affiliations:** 1 Top Institute Food and Nutrition, P.O. Box 557 6700 AN, Wageningen, the Netherlands; 2 Neuroimaging Center Groningen, University Medical Center Groningen, University of Groningen, Antonius Deusinglaan 2, 9713 AW, Groningen, the Netherlands; 3 Department of Experimental Psychology, University of Groningen, Grote Kruisstraat 2/1, 9712 TS, Groningen, the Netherlands; University of Akron, UNITED STATES

## Abstract

Increasingly consumption of healthy foods is advised to improve population health. Reasons people give for choosing one food over another suggest that non-sensory features like health aspects are appreciated as of lower importance than taste. However, many food choices are made in the absence of the actual perception of a food’s sensory properties, and therefore highly rely on previous experiences of similar consumptions stored in memory. In this study we assessed the differential strength of food associations implicitly stored in memory, using an associative priming paradigm. Participants (N = 30) were exposed to a forced-choice picture-categorization task, in which the food or non-food target images were primed with either non-sensory or sensory related words. We observed a smaller N400 amplitude at the parietal electrodes when categorizing food as compared to non-food images. While this effect was enhanced by the presentation of a food-related word prime during food trials, the primes had no effect in the non-food trials. More specifically, we found that sensory associations are stronger implicitly represented in memory as compared to non-sensory associations. Thus, this study highlights the neuronal mechanisms underlying previous observations that sensory associations are important features of food memory, and therefore a primary motive in food choice.

## Introduction

In a supermarket individuals are confronted with a lot of different food products to choose from. Food companies anticipate on this decision process by marketing their products not only by directing the attention of potential consumers towards the sensory properties of a product (i.e. taste, smell and texture), but also by highlighting additional features of a food product related to specific non-sensory related characteristics of these products. Examples of the latter characteristics are the time a product most likely is consumed (e.g., *breakfast* drink) and the health benefits of that particular product (e.g., *low calorie* drink). Such marketing strategies are supported by scientific findings indicating that food choice is indeed based on a broad range of product characteristics, including sensory and non-sensory related [1]. Recent studies focused on the mediating role of implicit associations on consumer choice [2–4]. However, the relative importance of each of these factors implicitly affecting food choice remains elusive. Therefore, we set up the current study to differentiate between the association strength of different food product characteristics.

Many food choices are made in the absence of the actual perception of a food’s sensory properties. Therefore, real life food choices highly rely on previous experiences of consumption of food products, which are stored in our memory system. The sight of food products triggers these memory traces. For example, when people are faced with or question to think of a food product, they have expectations about its taste based on memory from previous experiences with that particular or similar food product. According to the recognition heuristic of decision making, consumer choices are largely based on the recognition. This means that consumers prefer a match between their previous experiences (i.e. activated memory traces) and the sight of the food product when making food choices [5,6].

Different associations affect food choice. First, descriptions of sensory properties, frequently applied in product marketing, facilitate adequate recall of previous consumption (e.g., *sweet* apple) [7]. Second, alongside of taste characteristics, studies have also shown that food choice is influenced by associations with the context in which a food product was previously consumed. For example, when food is categorized as “for *breakfast*”, it is more preferred in the morning than in the afternoon, while food categorized as “for *dinner*”, is more preferred in afternoon than in the morning [8]. Furthermore, it was also found that people reported more post-meal hunger, for servings at inappropriate times [9]. Third, food choice is affected by health beliefs. For example, dieting people assigned greater importance to nutrition information available on food products when evaluating healthiness [10]. Analogous to this, it was found that people associated their healthy food choice with positive feelings as ways to promote well-being [11]. Altogether, these studies show that different associations affect food choice.

The extent to which these associations differentially affect food choice has recently attracted more attention. Taste was shown the most chosen as the first criterion in food choice [12]. A direct comparison between taste and health associations showed that the taste of an apple was indeed a stronger predictor of individual dessert choice as compared to its assigned health score [13]. Furthermore, food choices were guided by information about time of the consumption (i.e. “*breakfast* product”) only when people were yet unfamiliar with its taste [14]. Overall, taste associations seem to be the strongest predictor of food choice in case of familiar food products. However, the differential importance of taste associations with respect to health and context associations remains elusive.

Previous studies on food associations and food choice often used explicit behavioural measures of taste, health, or context associations. However, people are usually not or only to a limited extent aware that these learned associations affect the way they choose food products, namely through the generation of expectations [15]. In the present study, we directly compared taste, health, and context associations with food using an *associative priming paradigm*. In this paradigm, participants are asked to make decisions about a target image, preceded by a prime word. Because we were interested in association strength and not so much in semantic relation, it was chosen to present the primes *masked* [16]. The rationale behind this is that research has indicated that masked priming is affected by association strength whereas unmasked priming more by semantic relationship [17]. In our study we used words as primes activating either taste (i.e. *sweet* and *salty*), health (i.e. *healthy*, *unhealthy*), or context associations (i.e. *breakfast*, *dinner*). Pictures of food and non-food items were used as targets. It was expected that taste primes would facilitate the categorization of food target more than health and context related associations. Furthermore, the prime-target relation could be either congruent (i.e. in accordance) or incongruent. We hypothesized that people will respond faster on congruent trials than on incongruent trials because of facilitated processing of the target by the prime [18–23]. Participants responded significantly faster to strongly related pairs of (unconscious) prime words and target images of objects and animals compared to unrelated pairs [24]. In accordance with these findings, we assumed that stronger associations would benefit more (i.e. faster and more accurate responses) from the effect of priming [24–29].

Neuroscience adds value to understanding consumer decisions by unravelling the mechanism that are related to the observed choice [30]. Such process knowledge enables us to make inferences beyond existing explicit behavioural findings, since these behavioural responses reflect a single end-product of different processes involved in information processing. Previous studies have related specific event-related potentials (ERPs) [31] to associative memory processing. In the present study, we focus on the N400 component, found to be related to associative processing, because we consider this to be the stage of information processing where implicitly activated memory traces are compared to a perceived food product. It was, for example, found that the N400 amplitude was smaller in response to congruent compared to incongruent prime and target pairs [21,32,33]. On top of that, high compared to low associative strength between prime and target resulted in a smaller N400 amplitude, as well [34–38].

In sum, in the present study we used an associative priming paradigm in which participants identified food and non-food pictures preceded by food related prime words in order to examine implicit associations with food pictures. In addition to the behavioural measures we focussed on the N400 EEG component, reflecting associative memory.

## Material and Methods

### Participants

A total of 30 volunteers participated in the present study (15 males; M = 20 years, SD = 1 year). All the participants were recruited from at the faculty of Behavioral and Social Sciences of the University of Groningen. They were Caucasian, with good English language proficiency based on their participation in the English bachelor program of Psychology requiring English proficiency at the C1 level in the Common European Framework, roughly corresponding to TOEFL ITP sum score of at least 627, and a score 63 on the reading subscale. They had normal or corrected-to-normal vision. They had no history of an eating disorder or any other psychiatric, serious medical or neurological diseases. Also, none of the participants was on psychoactive or hypertensive medication. A total of four participants reported being vegetarian, and one participants reported being on a diet. Participants were given course credits in exchange for their participation. It was attempted to test all participants at least one hour after a meal, in the afternoon (at 14h or 15h). The local ethics committee of the faculty of Behavioral and Social Sciences of the University of Groningen reviewed and approved the present study. All participants signed informed consent.

### Apparatus

The participants were tested individually in a dimly lit, sound-attenuated room. The experiment was done on a personal computer running Windows 7, with a refresh rate of 60 Hz. The task was fully programmed in Matlab (The Mathworks, Inc. 2014), which was also used to collect the behavioral data.

EEG was recorded using 21 tin electrodes attached to an electrocap (ElektroCap International Inc., Eaton, Ohio, USA). The electrodes were placed according to the international 10–20 system. The amplifier was a REFA 8–72 (Twente-Medical Systems, Enschede, The Netherlands). An average reference was used. Sample frequency was 250 Hz. Two electrodes were placed at the mastoids and were used for off-line re-referencing of the EEG signal. An electrode placed on the sternum served as the participants ground. Four electrodes, placed at the left and right lateral canthi and above and below the right eye, were used to measure the Electro Oculogram (EOG). Data acquisition was performed using Brain Vision Recorder (version 1.03, BrainProducts GmbH, Munich, Germany).

### Stimuli

**Fig 1:**
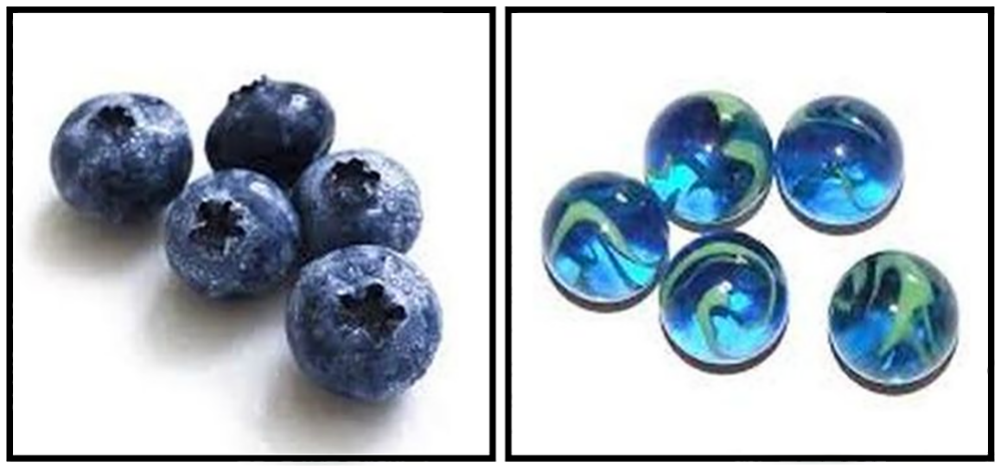
A pair of a matching food (left) and non-food (right) picture used as stimuli in this study. The two pictures were matched on color, size, orientation and amount.

In this study we used an associative priming paradigm. In this paradigm, the prime stimuli that were used were written words of either taste (i.e. *sweet* and *salty*), health (i.e. *healthy*, *unhealthy*), or context associations (i.e. *breakfast*, *dinner*). In addition, neutral primes (i.e. ‘XXXX’) of different lengths were used to control for the effect of word length of the different food related words. The independent variable for the primes was called *Modality*, and contained the levels ‘taste’, ‘health’, ‘context’, and ‘neutral’. The word categories of *Modality* were matched on word frequency using the online word frequency database of the Corpus of Contemporary American English (*taste*_*mean (sweet & salty)*_: 24.277, *health*_*mean (healthy & unhealthy)*_: 26.009, and context _*mean (breakfast & dinner)*_: 25.777). As targets we used pictures of food and non-food items that could be either congruent or incongruent to the prime. To investigate what combinations of food and modality are considered congruent in the population we conducted a pilot study. The independent variable for the relationship between the target and the prime was called *Congruence*, and could be either “congruent” or “incongruent”. All the pictures used in this study were derived from Google. All the pictures had a white background. Consequently, the non-food pictures were chosen to resemble the matching food picture (see Fig 1). Food and non-food pictures were matched on the color, size, orientation and amount of objects. There were in total six food categories (sweet, salty, breakfast, dinner, healthy, and unhealthy). For every category there were 15 food pictures and matching non-food pictures. Similarly, congruent and incongruent trials were equally balanced (i.e. 50/50). All the independent variables were within subject factors. μV/m^2^

### Task

**Fig 2:**
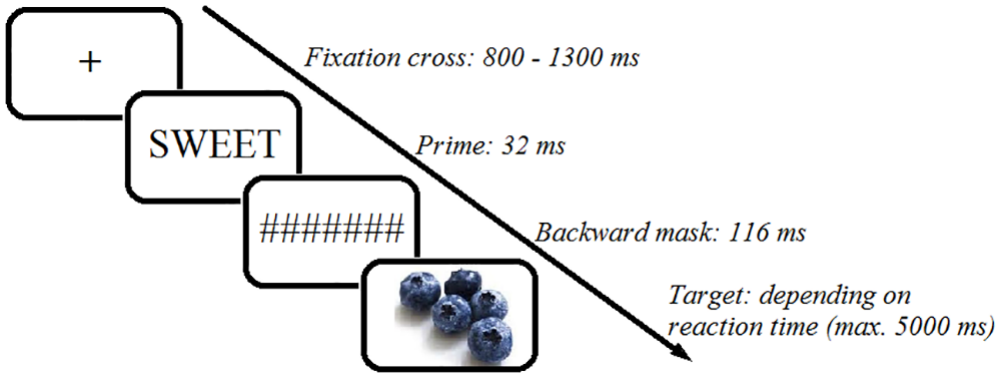
An overview of a trial. In this trial the prime word *sweet* is congruent with the food picture presented as target.

Each trial in the task began with a fixation cross (‘+’) varying between 800 and 1300 ms (see Fig 2). After the fixation cross, a prime word was shown for 32 ms. To reduce the visibility of the prime, a backward mask of nine hash tags (‘#########’) was presented for 116 ms after the prime. The target picture in the center of the screen followed the presentation of the mask and stayed on the screen until response, but with a maximum of 5000 ms. To investigate the effects of *Modality* and *Congruence* we measured the reaction time and accuracy to both food and non-food target pictures.

### Procedure

The participants were seated in one of the laboratory rooms facing a computer screen. They were first asked to fill out a questionnaire containing questions about their age, nationality, current feeling of hunger, diet, food restrictions, and if they were vegetarian. This questionnaire took approximately two minutes for the participants to complete. Thereafter, the main experiment started. During this part of the study, the participants were instructed to respond as quickly as possible if the presented picture contained either a food or a non-food object, by pressing a corresponding response mouse button with their left or right thumb. What button represented either food or non-food was counterbalanced between participants.

The task was divided in three blocks of 150 trials. The blocks differed in which prime modalities were assessed. In the first block the primes sweet, salty, breakfast, dinner, and neutral were assessed. In the second block; breakfast, dinner, healthy, unhealthy, and neutral primes were investigated. The third block focused on sweet, salty, healthy, unhealthy, and neutral primes. Within each block, 32 trials per prime and 22 neutral were given (total 150 trials per block). The order of the blocks was counterbalanced across participants and within each block the trials were randomized. Between the blocks, the participants were allowed to take a rest. The time of rest was not pre-set; participants rested approximately one minute. After completion of this task, the participants were instructed to wait for further instructions from the experimenter. The whole task took about eight minutes to complete.

After the associative priming task, craving for food was assessed. Explicit proxies of approach tendencies were collected for all food stimuli. Using visual analogue scales, food stimuli of the word-picture priming task were rated on liking at the moment of testing on a Visual Analogue Scale (VAS) using the question: “How much do you like this product?” which were answered on a scale (0–100) from “*don’t like*” (0) to “*like very much*” (100), which was answered on a scale (0–100) from *“not at all”* (0) to *“very much”* (100). Results are reported elsewhere. In total, the experiment took approximately 30 minutes. At the end of the experiment, the participants were debriefed.

### Data analysis

#### Behavioral data—reaction times

Due to technical issues, both behavioral and electrophysiological data of one participant was removed. Responses faster than 250 ms and slower than 1500 ms were excluded from the data analysis. Alongside these selection criteria, all incorrect trials (e.g., button press ‘food’ when a ‘non-food’ image is presented) were excluded from the analysis. Overall data selection resulted in the deletion of 929 observations (7% of total data). The data was fitted on linear mixed effect models with maximum likelihood (LME) using the *lme4* package [39] in the open source statistical language R (version 3.1.2) (R Core Team 2012). These models were chosen because they deal well with repeated measures and missing data.

Different models were build to test the effect of word length and priming effects of interest. Priming effects were analyzed for responses to food and non-food targets by a model including *Target* (two levels: food and non-food) and *Prime* (two levels: word (i.e. taste, health, context) and neutral) as fixed factors; the *Subjects* were used as a random factor (intercept). Furthermore, priming effects of interest were investigated in a separate model using responses to food targets, by entering *Modality* (three levels: taste, health, and context) and *Congruence* (based on prime and target combination; two levels: congruent and incongruent) as fixed factors; while taking into account inter-individual variability by adding *Subject* as a random factor (intercept). In order to control the latter priming effect for word length effects, the responses to food targets were compared between neutral primes of different length in a separate model including *Neutral* (four levels: 5, 6, 7, and 9 hashtags) as a fixed factor and *Subject* as a random factor. We report degrees of freedom, statistics, and p-values based on Satterthwaite’s approximations ANOVA. Statistical significance was evaluated at the 0.05 alpha level.

#### Electrophysiological data—ERP latencies and amplitudes

ERP data was processed using Brain Vision Analyzer 2 software (Brain Products, Munich, Germany). The EEG signal was filtered with a Butterworth high-pass filter of 0.5 Hz (24 dB/oct) and a low-pass filter of 15 Hz (24 dB/oct). Only correct trials with responses between 250 and 1500 ms were included for further analysis. The algorithm of Gratton, Coles, and Donchin (1983) was used to correct ocular movement artifacts [40]. Further artifact removal was applied by removing segments with an absolute difference larger than 200 μV or a voltage step per sampling point larger than 50 μV. Baseline correction was applied from -350 until target onset. Epochs were averaged starting 350 ms before target onset and lasting until 1500 ms post-target onset, separately for each *Target*, *Prime*, *Modality*, and *Congruence* level.

For confirmatory statistical analysis focused on the N400 priming effect, mean amplitudes (μV) were calculated in Brain Vision Analyzer using the amplitudes between 350–450 ms post-target onset from the average waveforms of individual participants. The averaged waveforms were grand averaged for display. Furthermore, we performed exploratory analysis on the frontoparietal electrodes based on previous evidence of priming [41–43]. Comparable to the analysis of reaction times, ERP amplitudes were analyzed by means of linear mixed effect models, using the *lme4* package (39). We report degrees of freedom, statistics, and p-values based on Satterthwaite’s approximations ANOVA. Statistical significance was evaluated at the 0.05 alpha level.

## Results

### Stronger focus on food compared to non-food images—N400 effect

The analysis of reaction times (i.e. time needed to classify a target as food or non-food) showed that participants responded faster to food compared to non-food targets, both preceded by a neutral prime (e.g., ‘XXXXX’) (*t*_*Target*_ (1869) = 2.34, p = .012).

**Fig 3:**
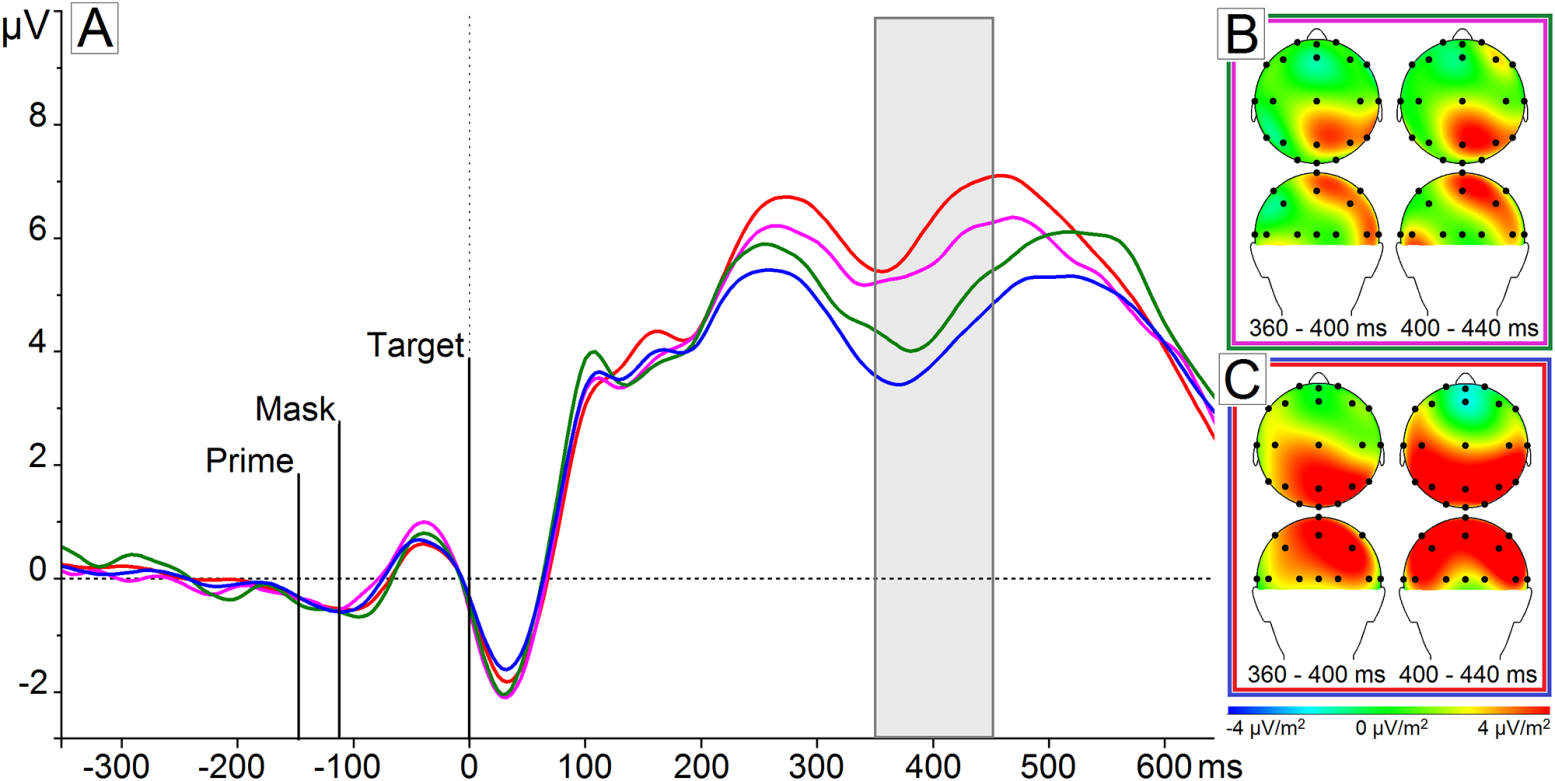
Scalp topographies and ERP: priming effect. The scalp topographies of the first task effect (B) indicate the mean difference between food target stimuli compared to non-food target stimuli following a neutral prime (e.g., ‘XXXX’) was most pronounced on the parietal electrodes Pz, P4, and P7. The scalp topographies of the second task effect (C) represent the mean different between food target stimuli compared to non-food target stimuli following a food-related prime (e.g., ‘sweet’ or ‘breakfast’) on all parietal electrodes. The ERPs of these effect locations combined into a parietal cluster (A) show a larger positive amplitude elicited by a food prime followed by a food target (red) as compared to a neutral prime followed by a food target (pink), a neutral prime followed by a non-food target (green), and a food prime followed by a non-food target (blue) subsequently between 350 and 450 ms.

Regarding the electrophysiological data corresponding to this effect, we observed that ERPs to food and non-food targets preceded by a neutral prime diverged clearly between 350 and 450 ms after target onset (*t*_*Target*_ (30) = -5.805, p < .001) (Fig 3a and 3c). This effect was most pronounced on the parietal electrodes (i.e. P4, P7, and Pz), based on visual inspection of the current source density (CSD) maps. Therefore, we conclude the presence of a smaller N400 peak in response to food compared to non-food target images.

### Priming with food-related words facilitates associative processing of food images

Interestingly, the effect described above was enhanced by the presentation of a word prime. Responses to food targets were faster following a word prime (e.g., ‘breakfast’) compared to a neutral prime (*t*_*Prime*_ (12580) = 4.745, p < .001), whereas responses to non-food target images preceded by a word prime were similar as responses to non-food target images preceded by a neutral prime (*t*_*Prime*_ (12580) = 1.524, NS). The electrophysiological data demonstrated that this priming effect was reflected in a smaller N400 amplitude in response to a food targets following a food prime (e.g., ‘sweet’ or ‘breakfast’) as compared to a neutral prime (e.g., ‘XXXX’) on the parietal electrodes within the interval of 350–450 ms (*t*_*Prime*_(90) = 2.70, p = .008) (Fig 3b and 3c). The priming effect was specific to food targets, since responses to non-food targets preceded by a word prime showed no difference in mean amplitude as compared to a neutral prime within this interval (*t*_*Prime*_ (90) = -0.43, NS). Thus, both behavioral and electrophysiological data showed priming of food associations.

### Taste associations stronger than health and context associations

Subsequently, the next prime effects of interest focused on the associations with different food characteristics, namely differentiation of *Modality* (i.e. ‘taste’, ‘health’, and ‘context’) preceding the presentation of food target. We observed that the priming effect (i.e. faster response food target images following food-related word prime compared to neutral prime) was stronger for ‘taste’ compared to ‘health’ (*t*_*Modality*: *taste-health*_ (5313) = 1.94, p = .05) and ‘context’ (*t*_*Modality*: taste-context_ (5313) = 4.75, p < .001). These effects were controlled for the effect of word length (*F*_*Neutral*_ (3,890) = 0.469, NS). Furthermore, the reaction time data revealed that the *Congruence* effect was different between primes (i.e. *Modality*) (*F*_*Congruence x Modality*_ (2,5313) = 6.20, p = .002). Remarkable, we observed that responses to congruent trials were slower as compared to incongruent trails for a ‘health’ prime (*t*_*Congruence*_ (5313) = -2.91, p = .003), and responses were not different between congruent and incongruent trials for ‘taste’ (*t*_*Congruence*_ (5313) = 1.22, NS) and ‘context’ (*t*_*Congruence*_ (5313) = 1.57, NS) primes.

**Fig 4:**
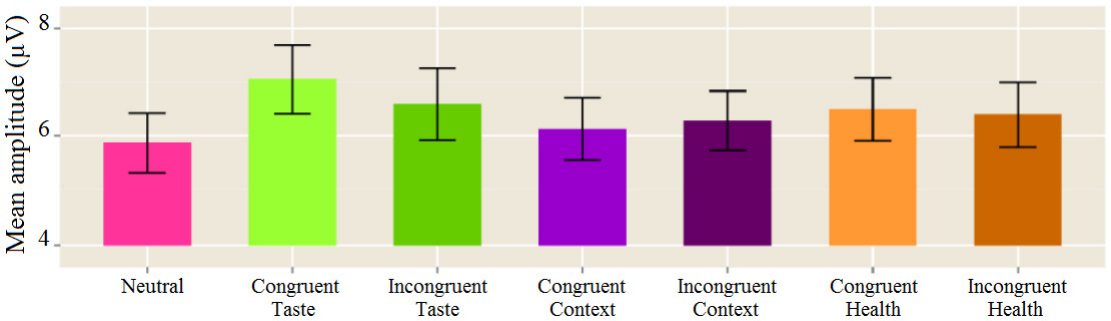
ERP amplitudes: priming effect. The mean and standard error of the mean ERP amplitudes in response to food target images averaged between 350 and 450 ms and combined into a parietal cluster (i.e. P3, P4, P7, P8, Pz). The bar graph shows 1) no differences between taste (green), time of consumption (purple), and health (orange) primes and 2) no interaction between congruence (congruent and incongruent respectively light and dark colored) and prime. The left (pink) bar represents the ERP amplitude following a neutral prime followed by a food target (see Fig 3c).

Compared to the reaction times, similar findings were observed in the electrophysiological data regarding the effect of *Modality* and *Congruence*. Results showed a smaller negative amplitude following a food target preceded by a taste prime compared to a context prime (*t*_*Modality*_ (150) = 3.24, p = .002) and a trend compared to a health prime (*t*_*Modality*_(150) = 1.95, p = .053). This effect was most pronounced at Pz, following visual inspection of the current source density (CSD) maps. In contrast to the reaction time data, however, the electrophysiological data did not show a differential *Congruence* effect for the different primes. N400 amplitudes were similar across congruent and incongruent pairs for taste, health, context as well as context primes on the parietal electrodes (*F*_*Congruence x Modality*_ (2,150) = 1.18, NS) (Fig 4).

### Food associations are frontally represented

**Fig 5:**
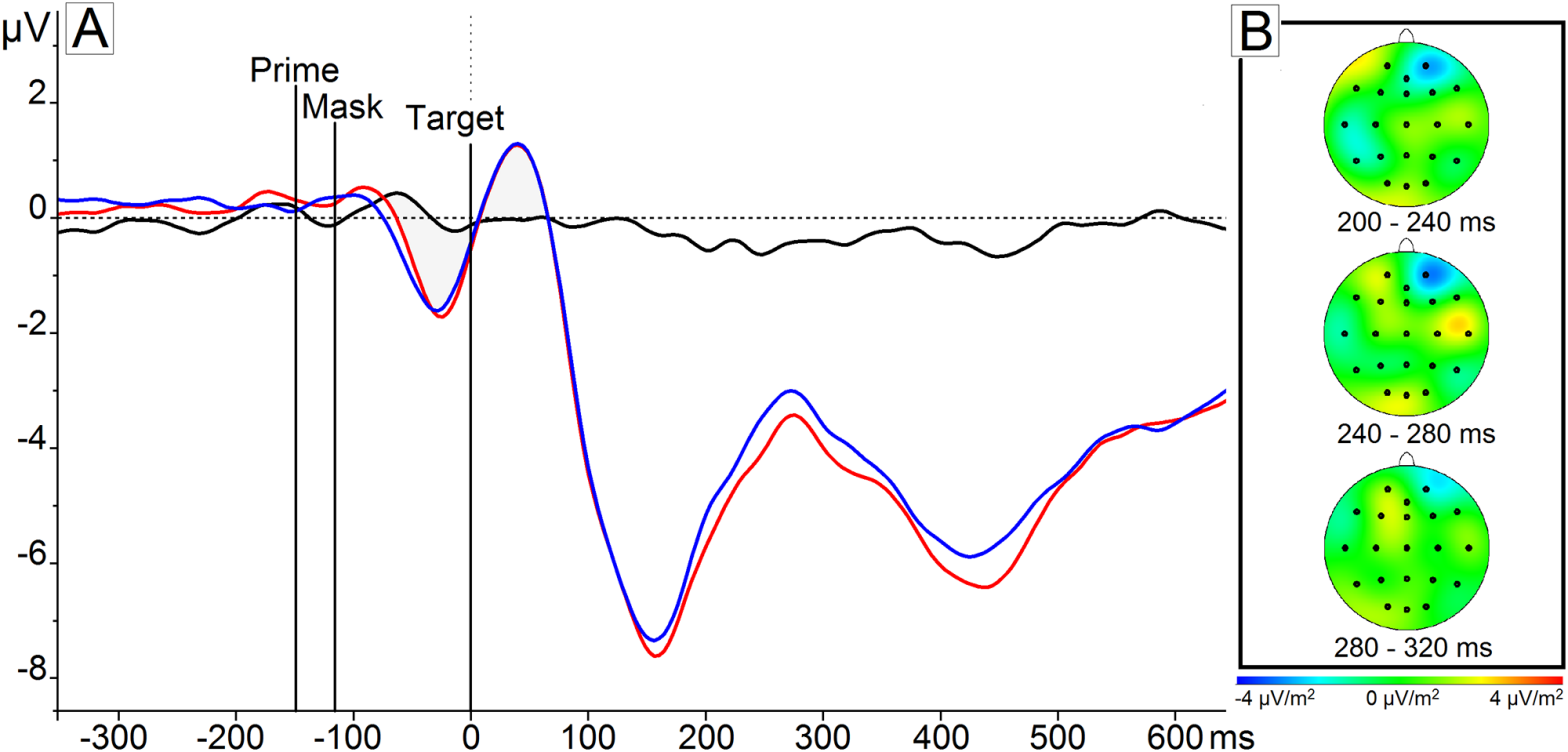
Scalp topographies and ERP: congruence effect. The scalp topographies of the congruence effect (B) indicate the mean difference between congruent food-related prime and food target pairs as compared to incongruent pairs on the frontal FP2 electrode. The ERPs on the frontal electrode (A) show a larger negative amplitude elicited by congruent food-related prime and food target pairs (red) as compared to incongruent pairs (blue) between 130 and 500 ms after target onset. The difference wave is represented in black.

Following the absence of an interaction between *Modality* and *Congruence* on the parietal electrodes, we explored other electrodes that have previously been described to reflect the congruence effect. Indeed, we observed a right lateralized frontal congruence effect (i.e. max at FP2 electrode) (Fig 5a). More specifically, congruent trials showed a larger negative amplitude starting 130 ms after target onset as compared to incongruent trials (*F*_*Congruence*_ (1,31) = 7.76, p = .009) (Fig 5b). This effect was not specific to any word prime (i.e. *Modality*) (*F*_*Congruence x Modality*_ (3,210) = .07, NS).

## Discussion

### General findings

In the present study we explored food associations by means of an associative priming paradigm. We consider the current differentiation between implicit food associations like taste, health, and context relevant in three ways. First, understanding the neural mechanisms of food memory provides us with insights for adequate marketing of food products. Second, we included taste, health, as well as context associations, which allowed us to make direct comparisons within one study. Finally, this study unraveled the implicit mechanisms of food associations, whereas previous studies on food associations have primarily used explicit behavioural measures. We focused on reaction times and EEG event-related potentials related to associative memory processing, namely the N400 at parietal electrodes, because previous studies showed this to be the stage of information processing where implicitly activated memory traces are compared to a perceived food product [21,32,44].

The current associative priming paradigm showed the plausibility to study factors affecting food choice without actual perception of the food product. Our findings reflected faster responses and a smaller parietal N400 peak in response to the sight of food compared to non-food. It has previously been shown that parietal neurons form bottom up “saliency maps” for quick selection of information in our environment [45–47,48]. More specifically, the event-related potential N400 was smaller in response to strongly salient items [49]. Based on our electrophysiological results, we consider that such “saliency maps” were constituted in the current paradigm by the repeated presentation of food information, thereby explaining enhanced processing of food compared to non-food images. These findings are in agreement with previous EEG studies suggesting increased motivational relevance and reinforcing properties of palatable food items to humans [50,51].

In addition to salience, the current brain potentials reflect the role of associative memory in food choice. Besides the bottom up “saliency maps”, shifts of attention are thought to depend on “top down” signals derived from a current activated memory traces (e.g., finding a *sweet* and/or *healthy* apple) [52]. The results showed that activating associations of taste, health, and context leads to faster processing and a smaller parietal N400 peak in response to food, whereas processing of non-food items was not affected by priming. Priming thereby implicitly facilitates food choice based on the sight of food products. The electrophysiological data revealed that priming elicits a state-dependent change in associative food memory [53].

Although there is increasing interest in how different factors are associated with food, how these “top down” signals differentially influence food choice remained elusive. For example, in a recent behavioral study on the use of simple descriptive food labels to promote healthy food choices, it was found that interventions that emphasize the taste of healthier foods are likely to be more effective at achieving healthier diets than those emphasizing health alone [13]. Furthermore, it has been suggested that extra attention should be paid to the tastefulness of healthy food products. Following the current neuroimaging study we consider that a shift from explicit behavioral methods towards implicit neuroimaging methods, provides us with a more valid and detailed understanding of the mechanisms underlying such food choices.

The current results point to the primary role of taste as a factor that directs consumers’ food choice. Thereby, we highlighted the potential role for associative food memory underlying the effects observed in behavioral consumer studies [54–56]. We conclude that the affined behavioural and electrophysiological results reflect a stronger association with food for taste compared to non-sensory factors [34].

In addition to priming studies showing enhanced processing for strong associations, it has been found that incongruent information can even hinder processing. In detail, high compared to low strength of associations resulted in a larger congruence effect, as reflected in a larger difference of N400 amplitudes at parietal electrodes [34–38]. However, we did not observe this interaction with congruence in addition to the effect of priming. Following adequate power and good categorization accuracy of the target images, according to the different primes in both our pilot as well as actual study participants, we speculate that more complex mechanisms underlie the effect of incongruent information in food choice.

It remains an ongoing challenge to facilitate healthy food choices. For example, it was found that health remains secondary to taste in the selection of corn chips [57]. In line with this, consumer willingness to compromise on taste for health in the specific case of the functional foods category was considered a risky strategic option [58]. Sensory aspects of food seems to be at the center of the development, maintenance and change of dietary patterns [59]. So, in order to control or even counteract the effect of the ‘tasty = unhealthy intuition’, healthy foods should be marketed to be (more) tasty [60]. It was suggested that efforts for promoting healthy eating behavior might benefit from an increasing attention towards memory principles in the development of interventions [59,61]. This study provides a starting point for studying how subtle differences in food associations affect food choice.

### Conclusion

The current study unraveled the implicit mechanism of food associations. We showed that taste associations are stronger related to food as compared to non-sensory associations like health and context. The modern day context of food choice requires more subtle choices among non-poisonous food products. This requires adequate “top-down” control using context associations in addition to the taste-conditioned approach-avoidance tendencies [62]. The current method of associative priming combined with electroencephalography is a suitable measure to study such subtle differences among these “top down” signals affecting food choice. The recurring importance of taste in choosing healthy foods suggests that these concepts and associations may be promising targets for future marketing interventions.
